# Prognostic implications of biventricular strain measurement in COVID‐19 patients by speckle‐tracking echocardiography

**DOI:** 10.1002/clc.23708

**Published:** 2021-08-06

**Authors:** Mohammad Khani, Sasan Tavana, Mohammadreza Tabary, Zahra Naseri Kivi, Isa Khaheshi

**Affiliations:** ^1^ Cardiovascular Research Center Shahid Beheshti University of Medical Sciences Tehran Iran; ^2^ Department of Pulmonary Medicine, Clinical Research and Development Center Shahid Modarres Hospital Tehran Iran; ^3^ Experimental Medicine Research Center Tehran University of Medical Sciences Tehran Iran

**Keywords:** COVID‐19, echocardiography, prognosis, speckle‐tracking echocardiography, strain

## Abstract

**Background:**

Recent reports have indicated the beneficial role of strain measurement in COVID‐19 patients.

**Hypothesis:**

To determine the association between right and left global longitudinal strain (RVGLS, LVGLS) and COVID‐19 patients' outcomes.

**Methods:**

Hospitalized COVID‐19 patients between June and August 2020 were included. Two‐dimensional echocardiography and biventricular global longitudinal strain measurement were performed. The outcome measure was defined as mortality, ICU admission, and need for intubation. Appropriate statistical tests were used to compare different groups.

**Results:**

In this study 207 patients (88 females) were enrolled. During 64 ± 4 days of follow‐up, 22 (10.6%) patients died. Mortality, ICU admission, and intubation were significantly associated with LVGLS and RVGLS tertiles. LVGLS tertiles could predict poor outcome with significant odds ratios in the total population (OR = 0.203, 95% CI: 0.088–0.465; OR = 0.350, 95% CI: 0.210–0.585; OR = 0.354, 95% CI: 0.170–0.736 for mortality, ICU admission, and intubation). Although odds ratios of LVGLS for the prediction of outcome were statistically significant among hypertensive patients, these odds ratios did not reach significance among non‐hypertensive patients. RVGLS tertiles revealed significant odds ratios for the prediction of mortality (OR = 0.322, 95% CI: 0.162–0.640), ICU admission (OR = 0.287, 95% CI: 0.166–0.495), and need for intubation (OR = 0.360, 95% CI: 0.174–0.744). Odds ratios of RVGLS remained significant even after adjusting for hypertension when considering mortality and ICU admission.

**Conclusion:**

RVGLS and LVGLS can be acceptable prognostic factors to predict mortality, ICU admission, and intubation in hospitalized COVID‐19 patients. However, RVGLS seems more reliable, as it is not confounded by hypertension.

## 1 INTRODUCTION

COVID‐19, caused by SARS‐CoV‐2, is a worldwide infectious viral disease initiating in December 2019 from Wuhan, China,^1^ and real‐time reverse‐transcriptase Polymerase Chain Reaction (RT‐PCR) test and similar microbiological methods are the gold standards of SARS‐CoV‐2 detection.^2^ Cardiovascular involvement is one of the main findings in COVID‐19 patients,^3^ including elevated cardiac biomarkers, heart failure, arrhythmias, myocarditis, and acute coronary syndrome.^4^ Transthoracic echocardiography (TTE) can be used as the initial imaging device to evaluate COVID‐19‐related cardiovascular manifestations, while it may also pave the way for clinical management.^5^

Speckle‐tracking echocardiography, a modality to measure myocardial strain, can be utilized as both a diagnostic and prognostic modality to objectively quantify myocardial deformation and dynamics.^6^ Recent reports have indicated the beneficial role of strain measurement in COVID‐19 patients.^7^, ^8^ To date, right ventricular longitudinal strain (RVLS) has been reported to be a powerful predictor of higher mortality in COVID‐19 patients.^9^ On the other hand, left ventricular global longitudinal strain (LVGLS) seems to decrease in these patients.^10^ Accordingly, regarding the lack of data on the prognostic value of biventricular strain measurement in COVID‐19 subjects, this study was performed to determine the association between right and left global longitudinal strain (RVGLS, LVGLS) and patients' outcomes, namely mortality, intensive care unit (ICU) admission, and need for intubation.

## 2 METHODS

### 2.1 Study design

**FIGURE 1:**
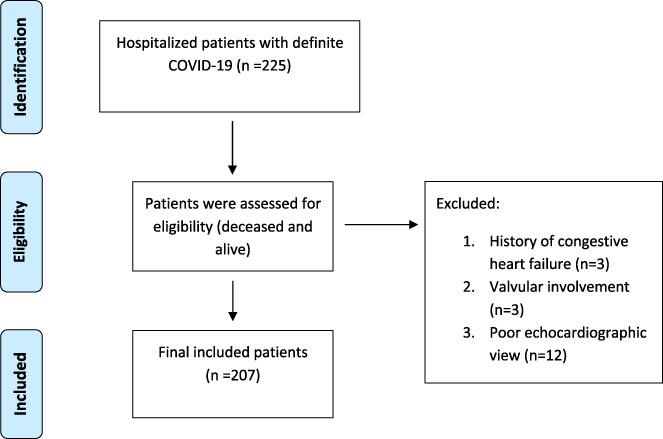
Study flow chart

In this prognostic prospective cohort study, 225 consecutive patients with established COVID‐19 were included; the diagnosis of COVID‐19 was made by real‐time RT‐PCR. Patients were consecutively enrolled from a general educational hospital (Shahid Modarres Hospital, Tehran, Iran) between June 21 and August 24, 2020. A consecutive series of COVID‐19 patients were included and underwent echocardiography in the first 12–24 h of admission. Furthermore, both deceased and survived cases were selected from a single center to reduce selection bias. The flow chart of patients' enrollment is depicted in Figure 1. Finally, 207 patients were included in our analyses. Clinical data were gathered by a predefined checklist including age, sex, outcome (mortality, ICU admission, and intubation), and background diseases (ischemic heart disease, chronic obstructive pulmonary disease, hypertension, asthma, hyperlipidemia, smoking, and diabetes mellitus). It should be maintained that all the study protocols were approved by the local ethics committee of Shahid Beheshti University of Medical Sciences.

### 2.2 Transthoracic echocardiography analysis

Two‐dimensional echocardiography was performed in all patients by a Siemens ACUSON SC‐2000 device based on the latest guidelines of the American Society of Echocardiography.^11^

The two‐dimensional echocardiography findings were left ventricular ejection fraction (LVEF), left ventricular end‐diastolic diameter (LVEDD), left ventricular end‐systolic diameter (LVESD), left atrial volume index (LAVI), interventricular septal diameter (IVSD), right ventricular end‐diastolic diameter (RVEDD), E', fractional area change (FAC), S velocity, tricuspid annular plane systolic excursion (TAPSE), tricuspid regurgitation peak velocity (TRPV), and systolic pulmonary artery pressure (PAP). LVEF was calculated based on the biplane Simpson method. Furthermore, LVEDD, LVESD, IVSD were calculated based on long‐axis parasternal view and M mode method. LV diastolic function was calculated by measuring E velocity, A velocity, LAVI, E', E/E', and E/A ratio.

The parameters of RVEDD, S velocity, TAPSE, RV FAC were used to evaluate the right ventricular function, and RVEDD was calculated in the 4‐chamber view. To clarify, RV FAC was obtained from the 4‐chamber view based on the following formula: (RVED area‐RVES area)/(RVED area*100). Tricuspid regurgitation peak gradient (TRPG) was also calculated based on the maximum gradient between RA & RV by the Doppler method. Finally, systolic PAP was obtained using the formula “TRPG+RA pressure” in which RA pressure was measured based on inferior vena cava (IVC) size and respiratory collapse of IVC.

LV global longitudinal strain (LVGLS) was calculated using three standard echocardiographic views, including 4‐chamber, 2‐chamber, and apical long‐axis based on the American Society of Echocardiography guideline. The region of interest (ROI) was set to be confined to the myocardium, and LVGLS, as bull's eye, was automatically calculated by the device (Figure S1). RVGLS was also calculated based on the 4‐chamber view, and RVGLS was automatically calculated by the device (Figure S2). Both measurements were performed in a 16‐segment model. It should be noted that the margin of the endocardium should be clearly defined for these evaluations and patients who did not meet this criterion were excluded from the study. All the measurements were performed by a single cardiologist specialized in this field (intra‐observer reliability: 0.90 [95% CI: 0.87–0.92]), and were adjusted for body surface area (BSA).

### 2.3 Statistical analysis

Data analysis were performed using the SPSS version 20.0 software. Kolmogorov–Smirnov test was utilized to test the normality of all data. Means of variables were compared using the appropriate statistical test. Also, outcome analysis was performed based on three tertiles for LVGLS and RVGLS. Odds ratios (OR) and 95% confidence intervals (CI) were calculated for the prediction of mortality, ICU admission, and need for intubation, and they were also adjusted for hypertension, where relevant, as the confounding effect of hypertension on LVGLS was described before.^12^ Since the number of patients with COPD was few, adjustment for COPD was not performed. Sensitivity was plotted for 1‐Specificity using ROC (receiver operating characteristic) curves for different cut‐off values of RVGLS, LVGLS, and outcome. The area under the ROC curve was used as an index to measure the test's predictive power. The *p*‐value for interaction was used to assess the interaction between hypertension and GLS. Whenever there was an interaction, a subgroup analysis was performed between hypertensive and non‐hypertensive patients. In case of no interaction, adjusted odds ratios were calculated. *p*‐values less than 0.05 were considered statistically significant.

## 3 RESULTS

In this study, 119 male (57.5%) and 88 female cases (42.5%) were enrolled. The mean age of patients was 54.5 ± 14.8 years. Patients' baseline characteristics are summarized in Table 1. The mean (standard deviation) systolic blood pressure, heart rate, and O2 saturation in the total population were 126.6 (19.9) mmHg, 85.0 (13.0) pulse per minute, and 89.7% (4.1), respectively. Laboratory findings (measured in the first 12–24 h of admission) are demonstrated in Table 2. Also, the mean O2 Sat was significantly lower in expired cases (*p* = 0.001), those with ICU admission (*p* = 0.001), and cases needing intubation (*p* = 0.001).

**TABLE 1:** Baseline characteristics of COVID‐19 patients

History	Total	Survived	Expired	*p*‐value
**Ischemic heart disease**	36 (17.4%)	31 (16.8%)	5 (22.7%)	.550
**Hypertension**	79 (38.2%)	64 (34.6%)	15 (68.2%)	**.002**
**Chronic obstructive pulmonary disease (COPD)**	2 (1.0%)	1 (0.5%)	1 (4.5%)	.202
**Asthma**	10 (4.8%)	10 (5.4%)	—	.604
**Smoking**	7 (3.4%)	7 (3.8%)	—	>0.999
**Hyperlipidemia**	24 (11.6%)	22 (11.9%)	2 (9.1%)	>0.999
**Diabetes mellitus**	52 (25.1%)	47 (25.4%)	5 (22.7%)	.784

**TABLE 2:** Laboratory test results among COVID‐19 cases

History	Total	Survived	Expired	*p*‐value
**Erythrocyte sedimentation rate (ESR)**	61.7 ± 25.6	61.0 ± 25.7	68.9 ± 24.2	.173
**D‐Dimer (ng/ml)**	469.2 ± 1204.4	469.1 ± 1144.62	469.5 ± 1379.1	.999
**Troponin (ng/ml)**	0.04 ± 0.18	0.04 ± .18	0.06 ± .14	.543
**Creatine phosphokinase (CPK) (IU/L)**	205.4 ± 269.6	196.1 ± 258.7	284.3 ± 345.4	.147
**Creatine phosphokinase MB (CK MB) (IU/L)**	25.0 ± 19.5	24.9 ± 19.5	26.1 ± 20.2	.783
**NT‐pro‐brain natriuretic peptide (pg/ml)**	1738.9 ± 5150.2	1525.8 ± 19.5	2525.5 ± 5235.1	.539

*Note*: Measurements were performed in the first 12–24 h of admission.

During 64 ± 4 days of follow‐up, 22 (10.6%) patients died. Among cases, 40 (19.3%) and 28 (13.5%) patients were admitted to intensive care unit (ICU) and Post‐ICU wards (patients with the intermediate condition), respectively. Intubation was also performed in 18 (8.7%) patients. Comparative results of echocardiography according to the outcome are shown in Table S1. LVEF, E', LAVI, RV FAC, TAPSE, and systolic PAP were contributing factors to patients' outcomes (*p* < 0.05). LVEF, E', RV FAC, TAPSE, were higher among patients with desirable outcome, while LAVI and systolic PAP were higher among those with poor outcome. Echocardiographic measurements were compared between three tertiles of LVGLS (Table S2) and RVGLS (Table S3). The tertiles for LVGLS and RVGLS are as follow: LVGLS (17.36–20.60), RVGLS (17.60–22.90). Regarding LVGLS, LVEF, LVEDD, E', LAVI, FAC, S velocity, and TAPSE revealed significant differences between the tertiles (*p* < 0.05). In terms of RVGLS, LVEF, LVEDD, E', LAVI, FAC, S Velocity, and TAPSE indicated significant differences according to the tertiles (*p* < 0.05).

**FIGURE 2:**
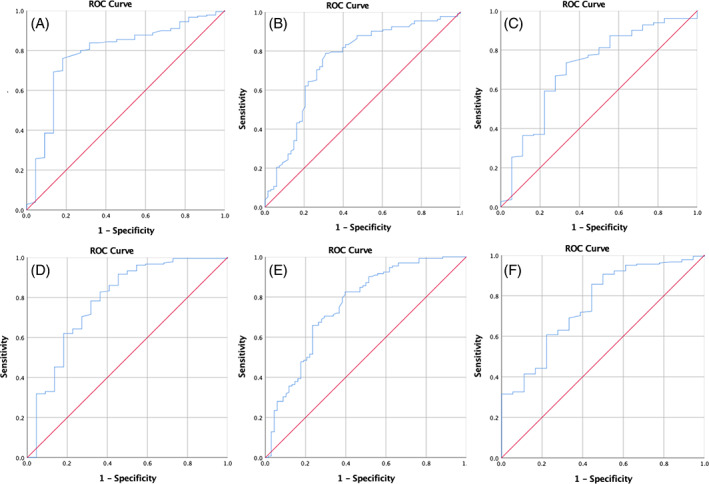
ROC curves for the sensitivity and specificity analysis of speckle tracking echocardiography. LVGLS: A. Mortality, B. ICU admission, C. Intubation. RVGLS: D. Mortality, E. ICU admission, F. Intubation

As demonstrated in Tables 3 and 4, mortality (*p* = .001, 0.001), ICU admission (*p* = .001, 0.001), and need for intubation (*p* = .006, 0.022) were significantly associated with LVGLS and RVGLS tertiles, respectively (lower measurement was associated with poor outcomes, ROC curves are presented in Figure 2, sensitivity and specificity analysis is summarized in Table 5). LVGLS tertiles could predict poor outcome with significant odds ratios for mortality (OR = 0.203, 95% CI: 0.088–0.465), ICU admission (OR = 0.350, 95% CI: 0.210–0.585), and need for intubation (OR = 0.354, 95% CI: 0.170–0.736). In the final model adjusted for hypertension and ischemic heart disease, LVGLS tertiles could predict mortality (OR = 0.203, 95% CI: 0.086–0.480), ICU admission (OR = 0.360, 95% CI: 0.213–0.610), and need for intubation (OR = 0.352, 95% CI: 0.163–0.761). As there was a significant interaction between hypertension and LVGLS tertiles (*p*‐value for interaction = 0.003, 0.016, and 0.003 for mortality, ICU admission, and intubation), a subgroup analysis was performed. Among non‐hypertensive patients, odds ratios of LVGLS for the prediction of outcome were not statistically significant (OR = 0.435, 0.515, 0.905; 95% CI: 0.153–1.237, 0.261–1.016, 0.305–2.687). However, these odds ratios were significant among hypertensive patients (OR = 0.090, 0.233, 0.174; 95% CI: 0.020–0.399, 0.100–0.542, 0.051–0.595).

**TABLE 3:** Outcomes according to tertiles for LVGLS

		LVGLS			
		Lower	Middle	Upper	*p*‐value
**Outcome**	**Survived**	51 (73.9%)	67 (97.1%)	67 (97.1%)	**.001**
	**Expired**	18 (26.1%)	2 (2.9%)	2 (2.9%)	
**Ward**	**ICU**	25 (36.2%)	10 (14.5%)	5 (7.2%)	**.001**
	**Post‐ICU**	15 (21.7%)	7 (10.1%)	6 (8.7%)	
	**Routine**	29 (42.0%)	52 (75.4%)	58 (84.1%)	
**Intubation**	**Yes**	12 (17.4%)	4 (5.8%)	2 (2.9%)	**.006**
	**No**	57 (82.6%)	65 (94.2%)	67 (97.1%)	

Abbreviations: ICU, intensive care unit; LVGLS, left ventricular global longitudinal strain.

**TABLE 4:** Outcomes according to tertiles for RVGLS

		RVGLS			
		Lower	Middle	Upper	*p*‐value
**Outcome**	**Survived**	53 (76.8%)	65 (94.2%)	67 (97.1%)	**.001**
	**Expired**	16 (23.2%)	4 (5.8%)	2 (2.9%)	
**Ward**	**ICU**	28 (40.6%)	7 (10.1%)	5 (7.2%)	**.001**
	**Post‐ICU**	14 (20.3%)	8 (11.6%)	6 (8.7%)	
	**Routine**	27 (39.1%)	54 (78.3%)	58 (84.1%)	
**Intubation**	**Yes**	11 (15.9%)	5 (7.2%)	2 (2.9%)	**.022**
	**No**	58 (84.1%)	64 (92.8%)	67 (97.1%)	

Abbreviations: ICU, intensive care unit; RVGLS, right ventricular global longitudinal strain.

**TABLE 5:** Sensitivity‐Specificity analysis for the relationship between LVGLS and RVGLS with mortality, ICU admission, and intubation

GLS‐outcome	AUC	*p*‐value	Sensitivity	Specificity	Cut‐off
**LVGLS**					
**Mortality**	0.785	<0.001	0.80	0.73	16.91
**ICU admission**	0.747	<0.001	0.79	0.66	17.58
**Intubation**	0.709	0.003	0.73	0.67	17.04
**RVGLS**					
**Mortality**	0.783	<0.001	0.83	0.64	14.88
**ICU admission**		<0.001	0.71	0.66	18.32
**Intubation**		<0.001	0.63	0.72	18.25

Abbreviations: ICU, intensive care unit; RVGLS, right ventricular global longitudinal strain.

Also, RVGLS tertiles revealed significant odds ratios for the prediction of mortality (OR = 0.322, 95% CI: 0.162–0.640), ICU admission (OR = 0.287, 95% CI: 0.166–0.495), and need for intubation (OR = 0.360, 95% CI: 0.174–0.744). In the final model adjusted for hypertension and ischemic heart disease, RVGLS tertiles could predict mortality (OR = 0.338, 95% CI: 0.167–0.681), ICU admission (OR = 0.291, 95% CI: 0.166–0.509), and need for intubation (OR = 0.358, 95% CI: 0.169–0.757). Among patients, there were not any significant interactions between hypertension and RVGLS tertiles for mortality and ICU admission (*p*‐value for interaction = 0.259, 0.345). However, hypertension and RVGLS tertiles interacted for the outcome of intubation (*p*‐value for interaction = 0.031). Thus, odds ratios of RVGLS were adjusted for hypertension when considering mortality and ICU admission, and they remained significant (OR = 0.365, 0.310;95% CI: 0.184–0.725, 0.180–0.536). A subgroup analysis was performed for the outcome of intubation; among hypertensive patients, the odds ratio of RVGLS for the prediction of intubation was significant (OR = 0.321, 95% CI: 0.119–0.863), while it revealed insignificant results among non‐hypertensive patients (OR = 0.619, 95% CI: 0.196–1.956).

## 4 DISCUSSION

This is one of the largest COVID‐19 populations studied by 2D strain echocardiography. In this cohort, it was found that the majority of findings in two‐dimensional echocardiography are related to the outcomes in COVID‐19 patients. Both LVGLS and RVGLS could predict all outcomes including ICU admission, need for intubation, and mortality in admitted COVID‐19 cases, even when adjusted for hypertension and ischemic heart diseases.

Our findings for RVGLS were congruent with the study of Krishnamoorthy et al.^13^ that showed right ventricular strain in COVID‐19 patients could be a prognostic factor, being lower in those with a poor prognosis. Nevertheless, they found no significant association for LVGLS. This difference can be attributable to their small sample size, which was limited to 12 cases. On the other hand, after excluding hypertensive patients, we found nonsignificant odds ratios for the association between LVGLS and outcomes. This may show the confounding effect of hypertension on LVGLS, as described by previous studies.^12^ At the same time, Baykan et al. indicated RVGLS and LVGLS as independent predictors of in‐hospital mortality among COVID‐19 patients.^14^

Although Cau and colleagues^15^ reported the best prognostic role of cardiac imaging methods in those with underlying cardiovascular disease, we excluded cases with valvular involvement and congestive heart failure to reduce possible bias.

A recent study by Li et al.^9^ evaluated 120 COVID‐19 cases and found RVGLS as a powerful predictor of poor outcomes (higher mortality). They suggested the application of RVGLS to identify COVID‐19 patients with a higher risk of mortality. Consistent with our findings, TAPSE and FAC were also related to RVGLS. On the other hand, a study by Schiavon et al.^16^ declared that the results of the aforementioned study by Li et al.^9^ were not sufficiently evidence‐based. They mentioned that proposed cutoff values for right ventricular longitudinal strain were within the normal range in healthy subjects and assumed that the active phase of the COVID‐19 infection leads to a form of “overload” of the right ventricle; this inability to overcompensate carries a worse prognosis. For this matter, in our study, the categorization was performed based on GLS tertiles.

Fukui et al.^17^ were led to believe that studies on all cardiac function parameters might explore some other prognostic factors in COVID‐19 patients. In our study, due to the larger sample size, the latent predictive roles for numerous echocardiographic parameters including LVEF, E', LAVI, FAC, TAPSE, and systolic PAP were revealed.

Monitoring of cardiovascular involvement is another indispensable part of the clinical management of COVID‐19 patients. Cameli et al.^18^ maintained that echocardiography is useful to discern between COVID‐19‐related myocardial damage and primary cardiac disease as well as to evaluate and monitor cardiovascular complications of COVID‐19, including acute myocarditis, arrhythmias, heart failure, sepsis‐induced myocardial impairment, and right ventricular failure resulting from high‐pressure mechanical ventilation.

We also found no association between the troponin level and any of RVGLS, LVGLS, or patients' outcomes. Consistent with this finding, Kocas et al.^19^ reported no significant association between troponin level and RVGLS. It may demonstrate the potential indirect effect of SARS‐CoV‐2 on cardiac function.

Finally, there is still a question: Why to use GLS measurements over routine echocardiographic parameters such as LVEF? First, GLS is considered to be a more reproducible method regardless of echocardiographic training as it has better inter‐ and intra‐observer reproducibility.^20^ Also, Strain echocardiography may reveal myocardial dysfunction before significant changes in ejection fraction and cardiac output.^21^ Strain measurement can also be valuable in detecting acute myocarditis,^22^ which may increase COVID‐19 mortality.^23^ Considering all the above‐mentioned rationales and the findings of our study, strain echocardiography can be a valuable tool for the prediction of outcome in hospitalized COVID‐19 patients.

This study was limited by the fact that only hospitalized COVID‐19 patients were included. Also, patients' pharmacologic therapy was not included in our analysis. Thus, further prospective studies are needed to take all confounding factors into account.

In conclusion, RVGLS and LVGLS can be both acceptable prognostic factors to predict mortality, ICU admission, and intubation in hospitalized COVID‐19 patients. However, RVGLS seems more reliable, as it is not confounded by hypertension. Also, other echocardiographic findings, such as LVEF, E', LAVI, FAC, TAPSE, and systolic PAP may be used as prognostic factors, if speckle tracking echocardiography is not available.

## CONFLICT OF INTEREST

The authors declare no potential conflict of interest.

## Supporting information


Appendix S1. Supporting information
The method of strain assessment and detailed measurements of echocardiographic parameters are available online.

## Data Availability

The data that support the findings of this study are available from the corresponding author upon reasonable request.
