# Supplementary material for: Prognostic implications of biventricular strain measurement in COVID‐19 patients by speckle‐tracking echocardiography
Source: Clin Cardiol. 2021 Aug 6;44(10):1475–81. doi: 10.1002/clc.23708 (PMC8420186; doi:10.1002/clc.23708)
Supplement: Supplementary file 1 — Appendix S1. Supporting information The method of strain assessment and detailed measurements of echocardiographic parameters are available online. [file CLC-44-1475-s001.pdf]

Supplementary materials:

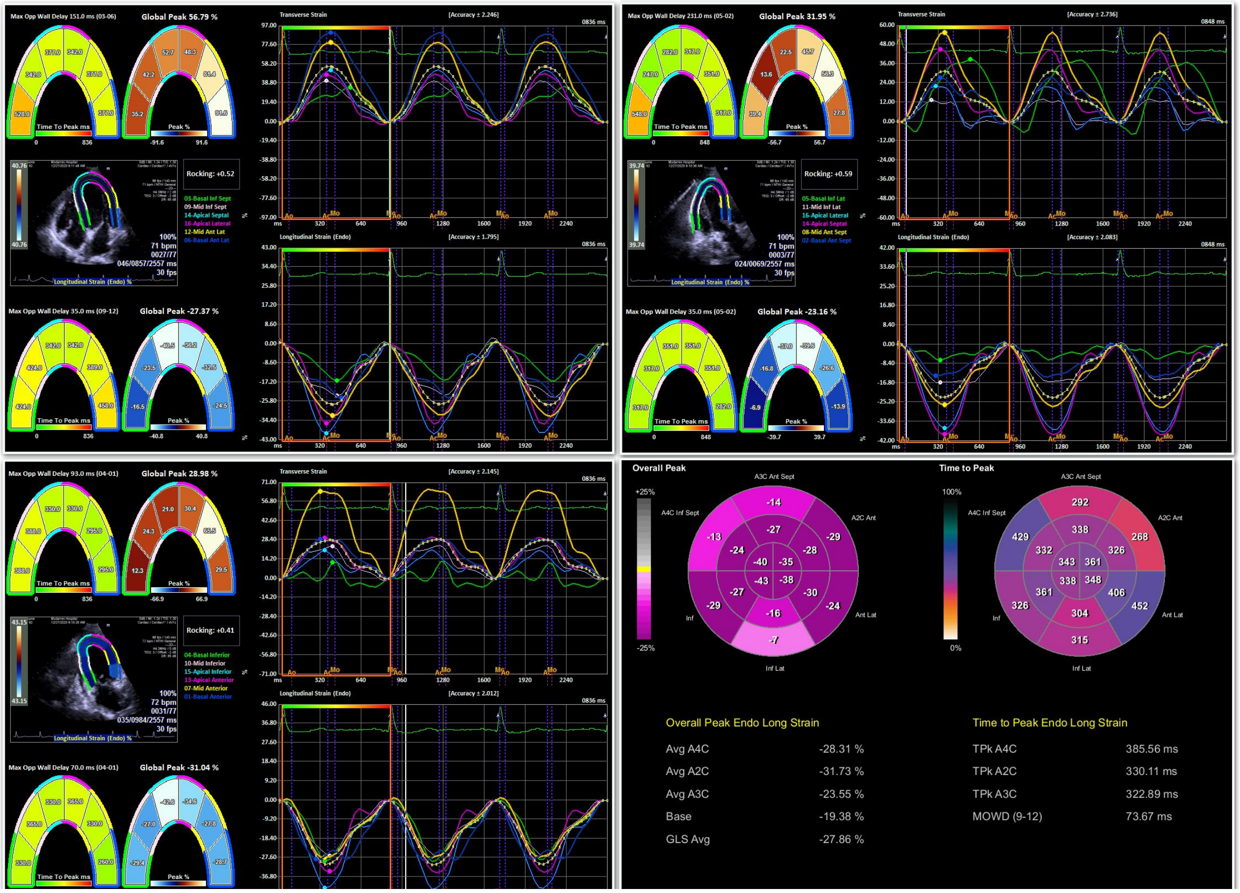

Supplementary Figure 1. Left ventricular endocardial margin was traced manually in 3 views (4-chamber, 3-chamber and 2-chamber). Then left ventricular global longitudinal strain (LVGLS) and bull's eye were automatically calculated by the device.

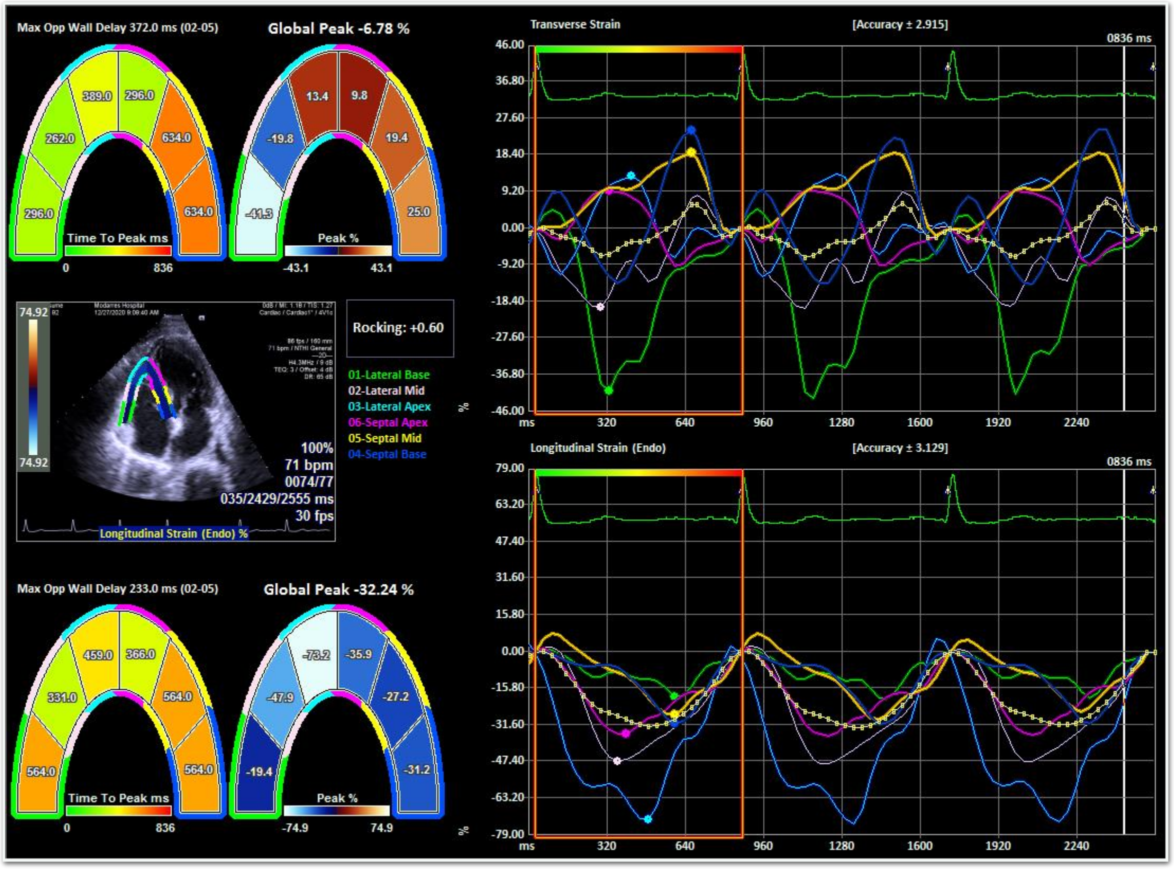

Supplementary Figure 2. Right ventricular endocardial margin was traced manually in the 4-chamber view. Then right ventricular global longitudinal strain (RVGLS) was automatically calculated by the device.

Supplementary Table 1- Echocardiographic measurements according to the outcome of COVID-19 patients

	Outcome					Ward							Intubation				
	Survived		Expired			ICU		Post-ICU		Routine			Yes		No		
	Mean	Standard Deviation	Mean	Standard Deviation	P Value	Mean	Standard Deviation	Mean	Standard Deviation	Mean	Standard Deviation	P Value	Mean	Standard Deviation	Mean	Standard Deviation	P Value
**LVEF**	59.35	9.10	51.77	10.48	**.001**	53.38	9.59	55.32	8.95	60.68	8.90	**.001**	54.89	7.87	58.89	9.61	.089
**LVEDD**	45.24	4.97	44.23	6.26	.383	44.68	5.80	45.25	5.27	45.24	4.91	.823	43.06	4.11	45.33	5.17	.072
**LVESD**	30.12	6.48	30.50	11.25	.813	30.95	8.93	30.54	5.49	29.86	6.82	.663	29.72	7.19	30.20	7.11	.785
**IVSD**	10.94	9.15	12.32	4.03	.486	11.33	3.36	13.34	15.08	10.56	8.09	.305	12.06	4.65	10.99	9.05	.624
**E/A**	1.12	.30	1.20	.66	.293	1.14	.52	1.05	.26	1.14	.31	.455	1.12	.32	1.13	.36	.920
**E'**	7.98	2.29	5.73	1.78	**.001**	6.20	1.77	7.11	2.27	8.31	2.28	**.001**	6.06	1.83	7.90	2.32	**.001**
**LAVI**	29.20	5.22	31.95	6.89	**.025**	30.63	6.10	30.07	6.17	29.05	5.11	.231	30.17	5.82	29.43	5.45	.586
**RVEDD**	28.12	14.72	31.00	10.50	.374	29.08	8.68	28.35	4.81	28.25	16.75	.950	31.67	11.51	28.12	14.56	.316
**RV FAC**	46.20	10.55	33.47	11.81	**.001**	36.99	11.57	42.44	11.65	47.59	10.08	**.001**	37.12	9.28	45.58	11.29	**.002**
**S Velocity**	12.91	2.32	12.18	3.06	.180	12.13	2.78	12.79	2.75	13.05	2.20	.101	12.06	2.44	12.91	2.41	.152
**TAPSE**	20.99	3.04	18.36	3.92	**.001**	18.98	3.65	19.75	3.57	21.41	2.80	**.001**	18.11	2.14	20.96	3.22	**.001**
**TRPV**	2.48	1.54	2.59	.40	.746	2.45	.41	2.51	.27	2.51	1.77	.971	2.61	.36	2.48	1.52	.738
**Systolic PAP**	28.45	6.68	41.82	14.85	**.001**	35.63	14.05	30.75	4.87	28.04	6.71	**.001**	40.67	14.26	28.84	7.51	**.001**

LVEF: left ventricular ejection fraction, LVEDD: left ventricular end-diastolic diameter, LVESD: left ventricular end-systolic diameter, IVSD: interventricular septal diameter, LAVI: left atrial volume index, RVEDD: right ventricular end-diastolic diameter, RV FAC: right ventricular fractional area change, TAPSE: tricuspid annular plane systolic excursion, TRPV: tricuspid regurgitation peak velocity, PAP: pulmonary artery pressure, ICU: Intensive Care Unit

Supplementary Table 2- Echocardiographic measurements in tertiles for LVGLS

		LVGLS Tertiles				
		**Lower**	**Middle**	**Upper**	**Total**	**P Value**
**LVEF**	**Mean**	52.19	59.22	64.22	58.54	**.001**
	**Std. Deviation**	8.92	7.69	7.86	9.52	
**LVEDD**	**Mean**	45.87	45.71	43.81	45.13	**.031**
	**Std. Deviation**	5.58	4.89	4.65	5.12	
**LVESD**	**Mean**	31.29	30.07	29.12	30.16	.197
	**Std. Deviation**	8.14	7.86	4.73	7.10	
**IVSD**	**Mean**	12.99	10.65	9.61	11.08	.066
	**Std. Deviation**	14.39	3.62	2.42	8.75	
**E/A**	**Mean**	1.09	1.09	1.19	1.12	.180
	**Std. Deviation**	.42	.34	.29	.35	
**E'**	**Mean**	7.00	7.58	8.64	7.74	**.001**
	**Std. Deviation**	2.41	2.17	2.16	2.34	
**LAVI**	**Mean**	30.96	29.65	27.87	29.49	**.004**
	**Std. Deviation**	6.42	4.75	4.70	5.47	
**RVEDD**	**Mean**	29.15	26.93	29.20	28.43	.571
	**Std. Deviation**	7.18	3.69	23.53	14.33	
**RV FAC**	**Mean**	40.61	45.04	48.89	44.84	**.001**
	**Std. Deviation**	12.02	10.74	9.81	11.36	
**S Velocity**	**Mean**	12.22	13.04	13.25	12.84	**.029**
	**Std. Deviation**	2.71	2.10	2.30	2.42	
**TAPSE**	**Mean**	19.49	21.32	21.33	20.71	**.001**
	**Std. Deviation**	3.58	2.76	3.00	3.23	
**TRPV**	**Mean**	2.71	2.42	2.35	2.50	.307
	**Std. Deviation**	2.38	.75	.35	1.46	
**Systolic PAP**	**Mean**	31.87	28.58	29.16	29.87	.068
	**Std. Deviation**	9.26	7.75	9.40	8.91	

LVGLS: left ventricular global longitudinal strain, LVEF: left ventricular ejection fraction, LVEDD: left ventricular end-diastolic diameter, LVESD: left ventricular end- systolic diameter, IVSD: interventricular septal diameter, LAVI: left atrial volume index, RVEDD: right ventricular end- diastolic diameter, RV FAC: right ventricular fractional area change, TAPSE: tricuspid annular plane systolic excursion, TRPV: tricuspid regurgitation peak velocity, PAP: pulmonary artery pressure, ICU: Intensive Care Unit

Supplementary Table 3- Echocardiographic measurements in tertiles for RVGLS

		RVGLS				
		**Lower**	**Middle**	**Upper**	**Total**	**P Value**
**LVEF**	**Mean**	53.96	58.13	63.54	58.54	.001
	**Std. Deviation**	10.13	7.37	8.41	9.52	
**LVEDD**	**Mean**	45.59	44.74	45.06	45.13	.614
	**Std. Deviation**	5.69	5.24	4.38	5.12	
**LVESD**	**Mean**	31.72	29.84	28.92	30.16	.060
	**Std. Deviation**	7.71	6.69	6.67	7.10	
**IVSD**	**Mean**	11.62	12.09	9.54	11.08	.192
	**Std. Deviation**	9.76	11.29	2.39	8.75	
**E/A**	**Mean**	1.09	1.12	1.16	1.12	.517
	**Std. Deviation**	.45	.31	.28	.35	
**E'**	**Mean**	6.84	7.78	8.59	7.74	.001
	**Std. Deviation**	2.48	2.04	2.17	2.34	
**LAVI**	**Mean**	30.58	30.14	27.75	29.49	.004
	**Std. Deviation**	6.24	5.17	4.50	5.47	
**RVEDD**	**Mean**	28.47	30.43	26.38	28.43	0.252
	**Std. Deviation**	7.54	23.41	3.05	14.33	
**RV FAC**	**Mean**	36.54	46.13	51.87	44.84	.001
	**Std. Deviation**	10.25	9.41	8.70	11.36	
**S Velocity**	**Mean**	12.10	13.12	13.29	12.84	.007
	**Std. Deviation**	2.50	2.50	2.08	2.42	
**TAPSE**	**Mean**	19.51	20.78	21.85	20.71	.001
	**Std. Deviation**	3.71	2.75	2.75	3.23	
**TRPV**	**Mean**	2.71	2.44	2.34	2.50	.297
	**Std. Deviation**	2.38	.36	.74	1.46	
**Systolic PAP**	**Mean**	31.78	29.91	27.91	29.87	.038
	**Std. Deviation**	9.18	8.11	9.11	8.91	

RVGLS: right ventricular global longitudinal strain, LVEF: left ventricular ejection fraction, LVEDD: left ventricular end-diastolic diameter, LVESD: left ventricular end- systolic diameter, IVSD: interventricular septal diameter, LAVI: left atrial volume index, RVEDD: right ventricular end- diastolic diameter, RV FAC: right ventricular fractional area change, TAPSE: tricuspid annular plane systolic excursion, TRPV: tricuspid regurgitation peak velocity, PAP: pulmonary artery pressure, ICU: Intensive Care Unit
